# (18-Crown-6-κ^6^ *O*)(pyrazolato-κ^2^ *N*,*N*′)-potassium(I)

**DOI:** 10.1107/S1600536808043237

**Published:** 2009-01-10

**Authors:** Kerstin Kunz, Hans-Wolfram Lerner, Michael Bolte

**Affiliations:** a Institut für Anorganische Chemie, Goethe-Universität Frankfurt, Max-von-Laue-Str. 7, 60438 Frankfurt/Main, Germany

## Abstract

The asymmetric unit of the title compound, [K(C_3_H_3_N_2_)(C_12_H_24_O_6_)], is composed of a potassium cation bonded to the six O atoms of a crown ether mol­ecule and the two N atoms of a pyrazolate anion. The K⋯O distances range from 2.8416 (8) to 3.0025 (8) Å, and the two K⋯N distances are 2.7441 (11) and 2.7654 (11) Å. The K cation is displaced by 0.8437 (4) Å from the best plane through the six O atoms. The latter plane is almost perpendicular to the plane of the pyrazolate ring [dihedral angle 83.93 (3)°].

## Related literature

For related literature on scorpionate complexes, see: Bieller *et al.* (2006 ▶); Morawitz *et al.* (2008 ▶); Trofimenko (1993 ▶).
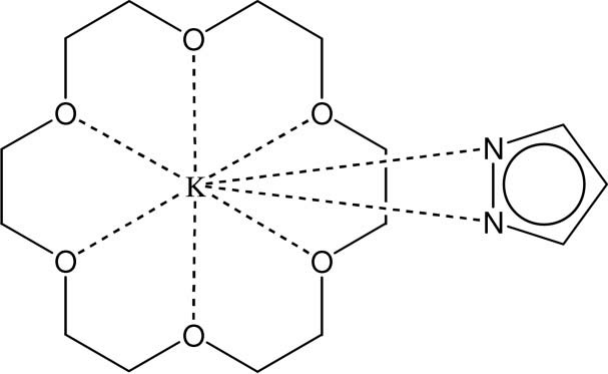


## Experimental

### 

#### Crystal data

[K(C_3_H_3_N_2_)(C_12_H_24_O_6_)]
*M* *_r_* = 370.49
Monoclinic, 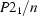

*a* = 11.5330 (6) Å
*b* = 8.2369 (5) Å
*c* = 20.7622 (10) Å
β = 101.612 (4)°
*V* = 1931.96 (18) Å^3^
*Z* = 4
Mo *K*α radiation
μ = 0.31 mm^−1^
*T* = 173 (2) K
0.25 × 0.12 × 0.12 mm

#### Data collection

STOE IPDS II two-circle-diffractometer
Absorption correction: multi-scan (*MULABS*; Spek, 2003 ▶; Blessing, 1995 ▶) *T*_min_ = 0.919, *T*_max_ = 0.966
23118 measured reflections
3602 independent reflections
3234 reflections with *I* > 2σ(*I*)
*R*_int_ = 0.034

#### Refinement

*R*[*F* ^2^ > 2σ(*F* ^2^)] = 0.026
*wR*(*F* ^2^) = 0.066
*S* = 1.03
3602 reflections
218 parameters
H-atom parameters constrained
Δρ_max_ = 0.18 e Å^−3^
Δρ_min_ = −0.20 e Å^−3^

### 

Data collection: *X-AREA* (Stoe & Cie, 2001 ▶); cell refinement: *X-AREA*; data reduction: *X-AREA*; program(s) used to solve structure: *SHELXS97* (Sheldrick, 2008 ▶); program(s) used to refine structure: *SHELXL97* (Sheldrick, 2008 ▶); molecular graphics: *XP* in *SHELXTL-Plus* (Sheldrick, 2008 ▶); software used to prepare material for publication: *SHELXL97*.

## Supplementary Material

Crystal structure: contains datablocks I, global. DOI: 10.1107/S1600536808043237/rz2281sup1.cif

Structure factors: contains datablocks I. DOI: 10.1107/S1600536808043237/rz2281Isup2.hkl

Additional supplementary materials:  crystallographic information; 3D view; checkCIF report

## Figures and Tables

**Fig. 1.**
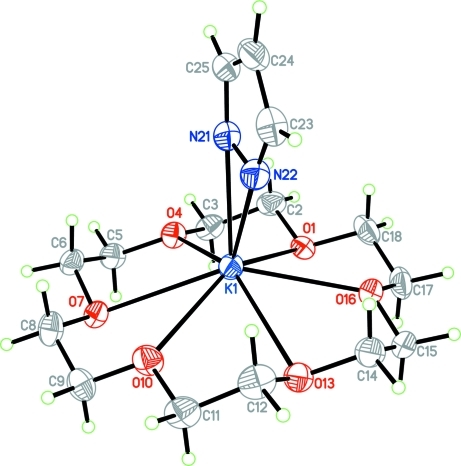
Perspective view of the title compound with the atom numbering scheme. Displacement ellipsoids are at the 50% probability level. H atoms are drawn as small spheres of arbitrary radii.

## supplementary crystallographic information

### Comment

We report here the X-ray crystal structure analysis of the potassium pyrazolide as complex with 18-crown-6, [K(18-crown-6)(C_3_H_3_N_2_)] or [K(18-crown-6)(pz)]. Following the first synthesis of a scorpionate complex, considerable progress has been made towards extending this area of chemistry (Trofimenko, 1993). Our studies have shown that an important factor influencing the stability of scorpionates appears to be the degree of steric crowding around the boron centre. The results of investigations in our group show that the scorpionates RB(3–*R*'pz)_3_^-^ and RB(4–*R*'pz)_3_^-^ decompose in the presence of transition metal salts much more easily when *R* and *R*' are bulky (Bieller *et al.*, 2006; Morawitz *et al.*, 2008). Now we have found that irradiation of Mn(C_5_H_3_)(Bpz_3_)_2_(THF)_2_ (I) in the presence of 18-crown-6 leads to the formation of potassium pyrazolide (II).

The asymmetric unit of the title compound, [C_12_H_24_O_6_]K^+^[C_3_H_3_N_2_]^-^, is composed of a potassium cation bonded to the six O atoms of a crown ether molecule and the two N atoms of a pyrazolate anion. The K···O distances range from 2.8416 (8) Å to 3.0025 (8) Å, and the two K···N distances are 2.7441 (11) Å and 2.7654 (11) Å. The K cation is displaced by 0.8437 (4) Å from the best plane through the six O atoms. The latter plane is almost perpendicular to the plane of the pyrazolate ring [dihedral angle 83.93 (3) °].

### Experimental

Mn(C_5_H_3_)(Bpz_3_)_2_(THF)_2_ (64 mg, 75.4 µmol) was dissolved in THF (25 ml). The solution was irradiated using an UV lamp (TQ 150, λmax = 510 nm) for 16 h, whereupon the colourless solution turned orange. After stirring overnight at ambient temperature the reaction mixture was treated with 18-crown-6 (20 mg, 81.2 µmol) and was stirred for 10 minutes. After a small amount of colourless precipitate had been removed by filtration, the clear filtrate was evaporated to dryness *in vacuo*. Single crystals of (II) were grown from a solution of (II) in THF at -35°C.

### Refinement

H atoms were geometrically positioned and refined using a riding model with fixed individual displacement parameters [U(H) = 1.2 *U*_eq_(C)] and with C_aromatic_—H = 0.95Å and C_methylene_—H = 0.99 Å.

### Crystal data

**Table:**

[K(C_3_H_3_N_2_)(C_12_H_24_O_6_)]	*F*(000) = 792
*M**_r_* = 370.49	*D*_x_ = 1.274 Mg m^−^^3^
Monoclinic, *P*2_1_/*n*	Mo *K*α radiation, λ = 0.71073 Å
Hall symbol: -P 2yn	Cell parameters from 23906 reflections
*a* = 11.5330 (6) Å	θ = 2.5–25.9°
*b* = 8.2369 (5) Å	µ = 0.31 mm^−^^1^
*c* = 20.7622 (10) Å	*T* = 173 K
β = 101.612 (4)°	Needle, colourless
*V* = 1931.96 (18) Å^3^	0.25 × 0.12 × 0.12 mm
*Z* = 4	

### Data collection

**Table:**

STOE IPDS II two-circle-diffractometer	3602 independent reflections
Radiation source: fine-focus sealed tube	3234 reflections with *I* > 2σ(*I*)
graphite	*R*_int_ = 0.034
ω scans	θ_max_ = 25.6°, θ_min_ = 2.7°
Absorption correction: multi-scan (*MULABS*; Spek, 2003; Blessing, 1995)	*h* = −12→14
*T*_min_ = 0.919, *T*_max_ = 0.966	*k* = −9→9
23118 measured reflections	*l* = −25→25

### Refinement

**Table:**

Refinement on *F*^2^	Secondary atom site location: difference Fourier map
Least-squares matrix: full	Hydrogen site location: inferred from neighbouring sites
*R*[*F*^2^ > 2σ(*F*^2^)] = 0.026	H-atom parameters constrained
*wR*(*F*^2^) = 0.066	*w* = 1/[σ^2^(*F*_o_^2^) + (0.036*P*)^2^ + 0.3751*P*] where *P* = (*F*_o_^2^ + 2*F*_c_^2^)/3
*S* = 1.03	(Δ/σ)_max_ = 0.001
3602 reflections	Δρ_max_ = 0.18 e Å^−^^3^
218 parameters	Δρ_min_ = −0.19 e Å^−^^3^
0 restraints	Extinction correction: *SHELXL*, Fc^*^=kFc[1+0.001xFc^2^λ^3^/sin(2θ)]^-1/4^
Primary atom site location: structure-invariant direct methods	Extinction coefficient: 0.0099 (8)

### Special details

**Table:**

Geometry. All e.s.d.'s (except the e.s.d. in the dihedral angle between two l.s. planes) are estimated using the full covariance matrix. The cell e.s.d.'s are taken into account individually in the estimation of e.s.d.'s in distances, angles and torsion angles; correlations between e.s.d.'s in cell parameters are only used when they are defined by crystal symmetry. An approximate (isotropic) treatment of cell e.s.d.'s is used for estimating e.s.d.'s involving l.s. planes.
Refinement. Refinement of *F*^2^ against ALL reflections. The weighted *R*-factor *wR* and goodness of fit *S* are based on *F*^2^, conventional *R*-factors *R* are based on *F*, with *F* set to zero for negative *F*^2^. The threshold expression of *F*^2^ > σ(*F*^2^) is used only for calculating *R*-factors(gt) *etc*. and is not relevant to the choice of reflections for refinement. *R*-factors based on *F*^2^ are statistically about twice as large as those based on *F*, and *R*- factors based on ALL data will be even larger.

### Fractional atomic coordinates and isotropic or equivalent isotropic displacement parameters (Å^2^)

**Table:**

	*x*	*y*	*z*	*U*_iso_*/*U*_eq_	
K1	0.50031 (2)	0.36001 (3)	0.173982 (11)	0.02711 (9)	
O1	0.70384 (7)	0.33603 (10)	0.10488 (4)	0.03149 (19)	
C2	0.72895 (12)	0.16959 (15)	0.09518 (6)	0.0375 (3)	
H2A	0.7929	0.1606	0.0699	0.045*	
H2B	0.6576	0.1149	0.0700	0.045*	
C3	0.76649 (11)	0.09050 (15)	0.16099 (6)	0.0341 (3)	
H3A	0.7879	−0.0243	0.1554	0.041*	
H3B	0.8366	0.1469	0.1866	0.041*	
O4	0.67091 (7)	0.09896 (9)	0.19483 (4)	0.02900 (18)	
C5	0.69922 (11)	0.02037 (14)	0.25736 (6)	0.0316 (3)	
H5A	0.7675	0.0750	0.2856	0.038*	
H5B	0.7209	−0.0943	0.2517	0.038*	
C6	0.59374 (11)	0.02826 (13)	0.28891 (6)	0.0312 (3)	
H6A	0.5233	−0.0153	0.2585	0.037*	
H6B	0.6078	−0.0382	0.3295	0.037*	
O7	0.57370 (7)	0.19353 (9)	0.30449 (4)	0.02792 (18)	
C8	0.48115 (11)	0.20382 (16)	0.34087 (6)	0.0369 (3)	
H8A	0.5059	0.1486	0.3838	0.044*	
H8B	0.4093	0.1489	0.3164	0.044*	
C9	0.45417 (11)	0.37812 (16)	0.35159 (6)	0.0358 (3)	
H9A	0.3904	0.3860	0.3770	0.043*	
H9B	0.5254	0.4330	0.3768	0.043*	
O10	0.41787 (7)	0.45411 (10)	0.28931 (4)	0.0337 (2)	
C11	0.38264 (12)	0.61956 (15)	0.29539 (6)	0.0373 (3)	
H11A	0.4487	0.6822	0.3219	0.045*	
H11B	0.3143	0.6243	0.3176	0.045*	
C12	0.34937 (11)	0.68958 (16)	0.22762 (7)	0.0390 (3)	
H12A	0.2892	0.6198	0.2000	0.047*	
H12B	0.3147	0.7989	0.2298	0.047*	
O13	0.45191 (7)	0.70035 (10)	0.19903 (4)	0.03146 (19)	
C14	0.42386 (12)	0.77471 (15)	0.13533 (6)	0.0396 (3)	
H14A	0.3977	0.8880	0.1394	0.047*	
H14B	0.3586	0.7147	0.1069	0.047*	
C15	0.53103 (13)	0.77270 (14)	0.10522 (6)	0.0397 (3)	
H15A	0.5160	0.8371	0.0641	0.048*	
H15B	0.5988	0.8219	0.1359	0.048*	
O16	0.55811 (8)	0.60970 (9)	0.09139 (4)	0.03225 (19)	
C17	0.65728 (12)	0.59892 (16)	0.05956 (6)	0.0390 (3)	
H17A	0.7291	0.6427	0.0887	0.047*	
H17B	0.6418	0.6632	0.0184	0.047*	
C18	0.67551 (12)	0.42399 (17)	0.04447 (6)	0.0396 (3)	
H18A	0.6026	0.3793	0.0167	0.047*	
H18B	0.7408	0.4138	0.0202	0.047*	
N21	0.32022 (9)	0.19783 (12)	0.09279 (5)	0.0333 (2)	
N22	0.27171 (9)	0.34696 (12)	0.10107 (5)	0.0347 (2)	
C23	0.16225 (12)	0.34869 (17)	0.06409 (6)	0.0405 (3)	
H23	0.1094	0.4381	0.0609	0.049*	
C24	0.13632 (13)	0.20214 (18)	0.03103 (6)	0.0444 (3)	
H24	0.0654	0.1709	0.0019	0.053*	
C25	0.23868 (13)	0.11318 (16)	0.05093 (6)	0.0387 (3)	
H25	0.2499	0.0057	0.0368	0.046*	

### Atomic displacement parameters (Å^2^)

**Table:**

	*U*^11^	*U*^22^	*U*^33^	*U*^12^	*U*^13^	*U*^23^
K1	0.02916 (14)	0.02496 (13)	0.02754 (13)	−0.00022 (9)	0.00652 (9)	0.00046 (9)
O1	0.0406 (5)	0.0316 (4)	0.0228 (4)	−0.0045 (4)	0.0075 (3)	−0.0025 (3)
C2	0.0438 (7)	0.0348 (6)	0.0376 (6)	−0.0046 (5)	0.0171 (5)	−0.0118 (5)
C3	0.0315 (6)	0.0282 (6)	0.0451 (7)	0.0019 (5)	0.0140 (5)	−0.0058 (5)
O4	0.0277 (4)	0.0273 (4)	0.0321 (4)	0.0044 (3)	0.0064 (3)	0.0016 (3)
C5	0.0347 (6)	0.0243 (5)	0.0333 (6)	0.0070 (5)	0.0008 (5)	0.0029 (4)
C6	0.0368 (6)	0.0217 (5)	0.0335 (6)	−0.0004 (5)	0.0030 (5)	0.0039 (4)
O7	0.0281 (4)	0.0248 (4)	0.0323 (4)	−0.0005 (3)	0.0096 (3)	0.0020 (3)
C8	0.0344 (6)	0.0399 (7)	0.0404 (7)	−0.0027 (5)	0.0172 (5)	0.0077 (5)
C9	0.0364 (7)	0.0442 (7)	0.0308 (6)	0.0010 (5)	0.0161 (5)	0.0006 (5)
O10	0.0379 (5)	0.0327 (4)	0.0324 (4)	0.0066 (3)	0.0114 (3)	−0.0026 (3)
C11	0.0365 (7)	0.0350 (6)	0.0440 (7)	0.0067 (5)	0.0166 (5)	−0.0078 (5)
C12	0.0308 (6)	0.0353 (6)	0.0506 (7)	0.0106 (5)	0.0076 (5)	−0.0033 (6)
O13	0.0326 (4)	0.0292 (4)	0.0309 (4)	0.0047 (3)	0.0022 (3)	0.0008 (3)
C14	0.0527 (8)	0.0266 (6)	0.0340 (6)	0.0080 (5)	−0.0041 (6)	0.0017 (5)
C15	0.0595 (8)	0.0254 (6)	0.0309 (6)	−0.0083 (6)	0.0010 (6)	0.0042 (5)
O16	0.0400 (5)	0.0275 (4)	0.0293 (4)	−0.0071 (3)	0.0070 (3)	0.0027 (3)
C17	0.0415 (7)	0.0466 (7)	0.0294 (6)	−0.0077 (6)	0.0086 (5)	0.0126 (5)
C18	0.0452 (7)	0.0526 (8)	0.0224 (5)	−0.0014 (6)	0.0104 (5)	0.0028 (5)
N21	0.0387 (6)	0.0322 (5)	0.0298 (5)	−0.0024 (4)	0.0092 (4)	0.0017 (4)
N22	0.0397 (6)	0.0325 (5)	0.0331 (5)	−0.0026 (4)	0.0099 (4)	0.0003 (4)
C23	0.0384 (7)	0.0470 (7)	0.0373 (7)	0.0047 (6)	0.0107 (5)	0.0084 (6)
C24	0.0434 (7)	0.0596 (9)	0.0271 (6)	−0.0151 (6)	−0.0002 (5)	0.0047 (6)
C25	0.0556 (8)	0.0352 (6)	0.0265 (6)	−0.0092 (6)	0.0107 (5)	−0.0017 (5)

### Geometric parameters (Å, °)

**Table:**

K1—N21	2.7441 (11)	C9—H9B	0.9900
K1—N22	2.7654 (11)	O10—C11	1.4349 (14)
K1—O16	2.8416 (8)	C11—C12	1.4976 (19)
K1—O10	2.8557 (8)	C11—H11A	0.9900
K1—O4	2.8877 (8)	C11—H11B	0.9900
K1—O13	2.9254 (8)	C12—O13	1.4286 (15)
K1—O1	2.9938 (9)	C12—H12A	0.9900
K1—O7	3.0025 (8)	C12—H12B	0.9900
K1—C12	3.5242 (12)	O13—C14	1.4341 (14)
O1—C2	1.4239 (15)	C14—C15	1.493 (2)
O1—C18	1.4282 (14)	C14—H14A	0.9900
C2—C3	1.4969 (18)	C14—H14B	0.9900
C2—H2A	0.9900	C15—O16	1.4207 (15)
C2—H2B	0.9900	C15—H15A	0.9900
C3—O4	1.4231 (14)	C15—H15B	0.9900
C3—H3A	0.9900	O16—C17	1.4343 (15)
C3—H3B	0.9900	C17—C18	1.498 (2)
O4—C5	1.4284 (13)	C17—H17A	0.9900
C5—C6	1.4946 (17)	C17—H17B	0.9900
C5—H5A	0.9900	C18—H18A	0.9900
C5—H5B	0.9900	C18—H18B	0.9900
C6—O7	1.4287 (13)	N21—C25	1.3405 (16)
C6—H6A	0.9900	N21—N22	1.3748 (15)
C6—H6B	0.9900	N22—C23	1.3391 (17)
O7—C8	1.4290 (14)	C23—C24	1.391 (2)
C8—C9	1.4951 (18)	C23—H23	0.9500
C8—H8A	0.9900	C24—C25	1.380 (2)
C8—H8B	0.9900	C24—H24	0.9500
C9—O10	1.4216 (14)	C25—H25	0.9500
C9—H9A	0.9900		
			
N21—K1—N22	28.90 (3)	C9—C8—H8B	109.7
N21—K1—O16	102.83 (3)	H8A—C8—H8B	108.2
N22—K1—O16	90.65 (3)	O10—C9—C8	108.63 (10)
N21—K1—O10	107.98 (3)	O10—C9—H9A	110.0
N22—K1—O10	91.60 (3)	C8—C9—H9A	110.0
O16—K1—O10	117.88 (2)	O10—C9—H9B	110.0
N21—K1—O4	97.94 (3)	C8—C9—H9B	110.0
N22—K1—O4	126.79 (3)	H9A—C9—H9B	108.3
O16—K1—O4	113.20 (2)	C9—O10—C11	112.02 (9)
O10—K1—O4	114.09 (2)	C9—O10—K1	123.81 (6)
N21—K1—O13	115.33 (3)	C11—O10—K1	118.66 (7)
N22—K1—O13	86.54 (3)	O10—C11—C12	107.85 (10)
O16—K1—O13	59.34 (2)	O10—C11—H11A	110.1
O10—K1—O13	58.88 (2)	C12—C11—H11A	110.1
O4—K1—O13	146.67 (2)	O10—C11—H11B	110.1
N21—K1—O1	104.07 (3)	C12—C11—H11B	110.1
N22—K1—O1	119.22 (3)	H11A—C11—H11B	108.4
O16—K1—O1	57.10 (2)	O13—C12—C11	109.71 (10)
O10—K1—O1	147.69 (2)	O13—C12—K1	54.11 (5)
O4—K1—O1	56.39 (2)	C11—C12—K1	87.18 (7)
O13—K1—O1	110.15 (2)	O13—C12—H12A	109.7
N21—K1—O7	111.90 (3)	C11—C12—H12A	109.7
N22—K1—O7	122.74 (3)	K1—C12—H12A	72.7
O16—K1—O7	144.82 (2)	O13—C12—H12B	109.7
O10—K1—O7	56.51 (2)	C11—C12—H12B	109.7
O4—K1—O7	57.65 (2)	K1—C12—H12B	160.8
O13—K1—O7	107.84 (2)	H12A—C12—H12B	108.2
O1—K1—O7	107.25 (2)	C12—O13—C14	110.96 (9)
N21—K1—C12	102.02 (3)	C12—O13—K1	102.58 (7)
N22—K1—C12	74.09 (3)	C14—O13—K1	105.13 (6)
O16—K1—C12	79.86 (3)	O13—C14—C15	109.23 (10)
O10—K1—C12	42.01 (3)	O13—C14—H14A	109.8
O4—K1—C12	153.02 (3)	C15—C14—H14A	109.8
O13—K1—C12	23.31 (3)	O13—C14—H14B	109.8
O1—K1—C12	133.39 (3)	C15—C14—H14B	109.8
O7—K1—C12	97.71 (3)	H14A—C14—H14B	108.3
C2—O1—C18	112.41 (9)	O16—C15—C14	109.22 (10)
C2—O1—K1	109.43 (6)	O16—C15—H15A	109.8
C18—O1—K1	108.93 (7)	C14—C15—H15A	109.8
O1—C2—C3	108.64 (9)	O16—C15—H15B	109.8
O1—C2—H2A	110.0	C14—C15—H15B	109.8
C3—C2—H2A	110.0	H15A—C15—H15B	108.3
O1—C2—H2B	110.0	C15—O16—C17	112.31 (9)
C3—C2—H2B	110.0	C15—O16—K1	118.32 (7)
H2A—C2—H2B	108.3	C17—O16—K1	122.61 (7)
O4—C3—C2	108.59 (10)	O16—C17—C18	108.32 (10)
O4—C3—H3A	110.0	O16—C17—H17A	110.0
C2—C3—H3A	110.0	C18—C17—H17A	110.0
O4—C3—H3B	110.0	O16—C17—H17B	110.0
C2—C3—H3B	110.0	C18—C17—H17B	110.0
H3A—C3—H3B	108.4	H17A—C17—H17B	108.4
C3—O4—C5	111.62 (9)	O1—C18—C17	108.73 (10)
C3—O4—K1	122.31 (6)	O1—C18—H18A	109.9
C5—O4—K1	119.86 (6)	C17—C18—H18A	109.9
O4—C5—C6	108.74 (9)	O1—C18—H18B	109.9
O4—C5—H5A	109.9	C17—C18—H18B	109.9
C6—C5—H5A	109.9	H18A—C18—H18B	108.3
O4—C5—H5B	109.9	C25—N21—N22	107.20 (10)
C6—C5—H5B	109.9	C25—N21—K1	175.53 (9)
H5A—C5—H5B	108.3	N22—N21—K1	76.41 (6)
O7—C6—C5	108.89 (9)	C23—N22—N21	107.35 (10)
O7—C6—H6A	109.9	C23—N22—K1	176.68 (9)
C5—C6—H6A	109.9	N21—N22—K1	74.69 (6)
O7—C6—H6B	109.9	N22—C23—C24	110.97 (12)
C5—C6—H6B	109.9	N22—C23—H23	124.5
H6A—C6—H6B	108.3	C24—C23—H23	124.5
C6—O7—C8	110.38 (9)	C25—C24—C23	103.12 (11)
C6—O7—K1	105.07 (6)	C25—C24—H24	128.4
C8—O7—K1	110.19 (7)	C23—C24—H24	128.4
O7—C8—C9	109.61 (9)	N21—C25—C24	111.35 (12)
O7—C8—H8A	109.7	N21—C25—H25	124.3
C9—C8—H8A	109.7	C24—C25—H25	124.3
O7—C8—H8B	109.7		
			
N21—K1—O1—C2	−57.90 (8)	O10—C11—C12—O13	−66.75 (13)
N22—K1—O1—C2	−84.52 (8)	O10—C11—C12—K1	−16.62 (9)
O16—K1—O1—C2	−154.25 (8)	N21—K1—C12—O13	−127.69 (7)
O10—K1—O1—C2	114.66 (8)	N22—K1—C12—O13	−120.20 (7)
O4—K1—O1—C2	32.36 (7)	O16—K1—C12—O13	−26.59 (6)
O13—K1—O1—C2	177.93 (7)	O10—K1—C12—O13	128.70 (8)
O7—K1—O1—C2	60.82 (7)	O4—K1—C12—O13	95.46 (8)
C12—K1—O1—C2	179.95 (7)	O1—K1—C12—O13	−4.80 (8)
N21—K1—O1—C18	65.36 (7)	O7—K1—C12—O13	117.87 (7)
N22—K1—O1—C18	38.75 (8)	N21—K1—C12—C11	115.40 (8)
O16—K1—O1—C18	−30.99 (7)	N22—K1—C12—C11	122.89 (8)
O10—K1—O1—C18	−122.08 (8)	O16—K1—C12—C11	−143.49 (8)
O4—K1—O1—C18	155.62 (8)	O10—K1—C12—C11	11.80 (7)
O13—K1—O1—C18	−58.81 (7)	O4—K1—C12—C11	−21.45 (11)
O7—K1—O1—C18	−175.92 (7)	O13—K1—C12—C11	−116.90 (11)
C12—K1—O1—C18	−56.79 (8)	O1—K1—C12—C11	−121.70 (7)
C18—O1—C2—C3	175.73 (10)	O7—K1—C12—C11	0.96 (8)
K1—O1—C2—C3	−63.09 (10)	C11—C12—O13—C14	−177.06 (10)
O1—C2—C3—O4	62.21 (12)	K1—C12—O13—C14	111.83 (9)
C2—C3—O4—C5	177.94 (9)	C11—C12—O13—K1	71.11 (10)
C2—C3—O4—K1	−29.89 (12)	N21—K1—O13—C12	58.90 (7)
N21—K1—O4—C3	101.35 (8)	N22—K1—O13—C12	56.37 (7)
N22—K1—O4—C3	103.29 (8)	O16—K1—O13—C12	149.19 (7)
O16—K1—O4—C3	−6.34 (8)	O10—K1—O13—C12	−37.60 (7)
O10—K1—O4—C3	−144.84 (8)	O4—K1—O13—C12	−124.72 (7)
O13—K1—O4—C3	−75.35 (9)	O1—K1—O13—C12	176.29 (7)
O1—K1—O4—C3	−0.30 (7)	O7—K1—O13—C12	−66.97 (7)
O7—K1—O4—C3	−147.70 (8)	N21—K1—O13—C14	−57.21 (8)
C12—K1—O4—C3	−121.14 (9)	N22—K1—O13—C14	−59.73 (7)
N21—K1—O4—C5	−108.68 (8)	O16—K1—O13—C14	33.09 (7)
N22—K1—O4—C5	−106.74 (8)	O10—K1—O13—C14	−153.70 (8)
O16—K1—O4—C5	143.63 (7)	O4—K1—O13—C14	119.17 (8)
O10—K1—O4—C5	5.13 (8)	O1—K1—O13—C14	60.18 (8)
O13—K1—O4—C5	74.62 (9)	O7—K1—O13—C14	176.92 (7)
O1—K1—O4—C5	149.67 (8)	C12—K1—O13—C14	−116.10 (10)
O7—K1—O4—C5	2.26 (7)	C12—O13—C14—C15	−175.93 (9)
C12—K1—O4—C5	28.83 (11)	K1—O13—C14—C15	−65.73 (10)
C3—O4—C5—C6	−177.84 (9)	O13—C14—C15—O16	67.33 (12)
K1—O4—C5—C6	29.22 (11)	C14—C15—O16—C17	177.37 (9)
O4—C5—C6—O7	−67.15 (11)	C14—C15—O16—K1	−30.65 (12)
C5—C6—O7—C8	−173.98 (9)	N21—K1—O16—C15	111.08 (8)
C5—C6—O7—K1	67.25 (9)	N22—K1—O16—C15	84.64 (8)
N21—K1—O7—C6	51.07 (7)	O10—K1—O16—C15	−7.51 (9)
N22—K1—O7—C6	81.39 (7)	O4—K1—O16—C15	−144.33 (8)
O16—K1—O7—C6	−119.30 (7)	O13—K1—O16—C15	−0.94 (8)
O10—K1—O7—C6	148.71 (7)	O1—K1—O16—C15	−150.32 (9)
O4—K1—O7—C6	−34.43 (6)	O7—K1—O16—C15	−78.07 (9)
O13—K1—O7—C6	178.94 (6)	C12—K1—O16—C15	10.94 (8)
O1—K1—O7—C6	−62.45 (7)	N21—K1—O16—C17	−99.97 (8)
C12—K1—O7—C6	157.38 (6)	N22—K1—O16—C17	−126.41 (8)
N21—K1—O7—C8	−67.83 (7)	O10—K1—O16—C17	141.43 (8)
N22—K1—O7—C8	−37.51 (8)	O4—K1—O16—C17	4.61 (9)
O16—K1—O7—C8	121.80 (7)	O13—K1—O16—C17	148.00 (9)
O10—K1—O7—C8	29.81 (7)	O1—K1—O16—C17	−1.38 (8)
O4—K1—O7—C8	−153.33 (8)	O7—K1—O16—C17	70.88 (9)
O13—K1—O7—C8	60.04 (7)	C12—K1—O16—C17	159.88 (8)
O1—K1—O7—C8	178.65 (7)	C15—O16—C17—C18	−177.58 (10)
C12—K1—O7—C8	38.48 (7)	K1—O16—C17—C18	31.82 (12)
C6—O7—C8—C9	−175.05 (10)	C2—O1—C18—C17	−176.81 (10)
K1—O7—C8—C9	−59.45 (11)	K1—O1—C18—C17	61.72 (11)
O7—C8—C9—O10	59.92 (13)	O16—C17—C18—O1	−62.76 (13)
C8—C9—O10—C11	176.08 (10)	O16—K1—N21—N22	−67.14 (6)
C8—C9—O10—K1	−30.63 (13)	O10—K1—N21—N22	58.19 (6)
N21—K1—O10—C9	106.21 (9)	O4—K1—N21—N22	176.78 (6)
N22—K1—O10—C9	130.46 (9)	O13—K1—N21—N22	−5.23 (7)
O16—K1—O10—C9	−137.95 (8)	O1—K1—N21—N22	−125.99 (6)
O4—K1—O10—C9	−1.51 (9)	O7—K1—N21—N22	118.53 (6)
O13—K1—O10—C9	−144.56 (9)	C12—K1—N21—N22	15.04 (6)
O1—K1—O10—C9	−66.20 (10)	C25—N21—N22—C23	0.02 (12)
O7—K1—O10—C9	1.40 (8)	K1—N21—N22—C23	177.28 (9)
C12—K1—O10—C9	−165.70 (10)	C25—N21—N22—K1	−177.26 (9)
N21—K1—O10—C11	−102.15 (8)	O16—K1—N22—N21	116.04 (6)
N22—K1—O10—C11	−77.90 (8)	O10—K1—N22—N21	−126.04 (6)
O16—K1—O10—C11	13.69 (9)	O4—K1—N22—N21	−3.98 (7)
O4—K1—O10—C11	150.13 (8)	O13—K1—N22—N21	175.27 (6)
O13—K1—O10—C11	7.09 (8)	O1—K1—N22—N21	64.07 (6)
O1—K1—O10—C11	85.44 (9)	O7—K1—N22—N21	−75.70 (6)
O7—K1—O10—C11	153.04 (9)	C12—K1—N22—N21	−164.70 (6)
C12—K1—O10—C11	−14.06 (8)	N21—N22—C23—C24	0.11 (14)
C9—O10—C11—C12	178.50 (10)	N22—C23—C24—C25	−0.18 (14)
K1—O10—C11—C12	23.70 (12)	C23—C24—C25—N21	0.19 (14)
